# Prevalence and patterns of antimicrobial resistance among *Escherichia coli* isolated from Zambian dairy cattle across different production systems

**DOI:** 10.1038/srep12439

**Published:** 2015-07-27

**Authors:** Geoffrey Mainda, Paul B. Bessell, John B. Muma, Sean P. McAteer, Margo E. Chase-Topping, James Gibbons, Mark P. Stevens, David L. Gally, Barend M. deC. Bronsvoort

**Affiliations:** 1 Divisions of Immunity and Infection, Veterinary Clinical Sciences; 2 Genetics and Genomics, The Roslin Institute and The Royal (Dick) School of Veterinary Sciences, The University of Edinburgh, Easter Bush, Midlothian, EH25 9RG, United Kingdom; 3 District Veterinary Office, Ministry of Agriculture and Livestock, P.O. Box 80285, Kabwe, Zambia; 4 Department of Disease Control, School of Veterinary Medicine, University of Zambia, Zambia; 5 Centre for Immunity, Infection and Evolution, Kings Buildings, University of Edinburgh, Charlotte Auerbach Road, Edinburgh, EH9 3FL, United Kingdom; 6 School of Veterinary Medicine, Veterinary Science Centre, Belfield, Dublin 4, Eire

## Abstract

This study focused on the use of antibiotics on small, medium and commercial-sized dairy farms in the central region of Zambia and its relationship to antibiotic resistance in *Escherichia coli*. A stratified random sample of 104 farms was studied, representing approximately 20% of all dairy farms in the region. On each farm, faecal samples were collected from a random sample of animals and a standardised questionnaire on the usage of antibiotics was completed. An *E. coli* isolate was obtained from 98.67% (371/376) of the sampled animals and tested for resistance to six classes of antibiotics. The estimated prevalence of resistance across the different farming systems was: tetracycline (10.61; 95%CI: 7.40–13.82), ampicillin (6.02; 95%CI: 3.31–8.73), sulfamethoxazole/ trimethoprim (4.49; 95%CI: 2.42–6.56), cefpodoxime (1.91; 95%CI: 0.46–3.36), gentamicin (0.89; 95%CI: 0.06–1.84) and ciprofloxacin (0%). Univariate analyses indicated certain diseases, exotic breeds, location, farm size and certain management practices as risk factors for detection of resistance, whereas multivariate analyses showed an association with lumpy skin disease and a protective effect for older animals (>25 months). This study has provided novel insights into the drivers of antibiotic use and their association with antibiotic resistance in an under-studied region of Southern Africa.

There is little information on the use of antibiotics and antimicrobial resistant (AMR) bacteria on farms in low income countries, where the majority of the livestock are kept on smallholdings^1^. A similar lack of information also exists for the commercial sectors in such countries. Knowledge of antibiotic use and the factors influencing the decision to use them is important for the management of bacterial livestock infectious diseases; the prudent use and good stewardship of antibiotics; monitoring of antibiotic residues in food of animal origin and limiting the selection of bacterial resistant to antimicrobials^2^.

In the United Kingdom (UK)/European Union (EU), antibiotics are sold as prescription-only medicines (POM), while, in sub-Saharan Africa, veterinary antimicrobials are readily sold in shops and markets without prescriptions with no formal centralised documentation of use^3^. Therefore, further information is required on the usage of different antibiotics in the livestock sectors of developing countries and levels of resistance need to be ascertained. It is important to determine if unrestricted use has led to high levels of resistance to certain antibiotics which may have an impact on the efficacy of treatments for local human infections.

The prevalence of antibiotic resistant bacteria may be an indicator of levels of antimicrobial use in livestock. Some studies have been carried out in Zambia^4^ and elsewhere^5^,^6^ but these were primarily in relation to specific diseases such as mastitis or diarrhoea. There is, however, little information on the prevalence of antimicrobial resistance from the commensal flora of the wider healthy population and how this may be associated with antibiotic use and other farm management practices.

Dairy farming in Zambia is an important growing livestock industry with support for smallholders coming from the government and Non-Governmental Organisations (NGOs). In addition, there are larger commercial operations that have very different practices and management, but which are also important for the economy. The smaller units generally contribute milk daily to co-operative collection centres for onward supply to processing or local sales. The primary objective of the present study was to determine the association between the prevalence of antibiotic resistant enteric *E. coli* in dairy cattle and the use of veterinary antibiotics^7^ by dairy farmers in Lusaka (capital city) and the surrounding districts in Zambia. In addition, we aimed to determine if there is appropriate stewardship of antibiotic use in this sector and identify potential ways to improve on usage practices. These results should inform the development of new policies and guidelines aimed at improving the rational use of antibiotics. The sampling also provides isolates and data which can be analysed for resistance mechanisms and for future comparisons with human isolate data.

## Results

### Distribution of the study farms

**Figure 1:**
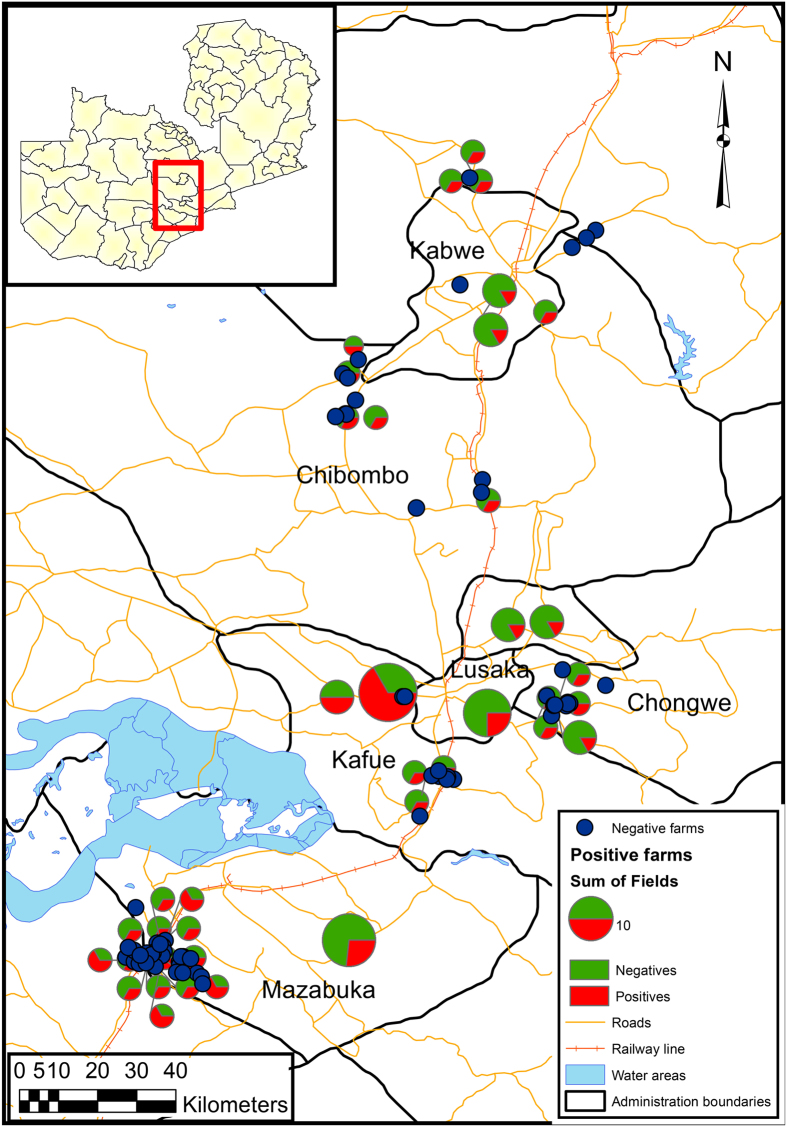
Map of Zambia (insert), depicting the location (red rectangle) of the study farms in the following Zambian districts; Kabwe, Chibombo, Chongwe, Lusaka, Kafue and Mazabuka. The pie charts indicate the relative sample sizes on each farm (ranging from the smallest chart representing 1 animal and the largest representing 18 animals. The legend shows an example of a pie chart representing 10 sampled animals and the red shading equals the proportion of animals that had a resistant isolate. Other features on the map include main roads (orange lines) and water areas (blue features). The map was generated in ARCGIS 10.1 software ARCGIS® (ESRI, ArcGIS desktop: release 10. Environmental Systems Research Institute, CA (2011)). The shape file was downloaded from Diva gis (http://www.diva-gis.org/gdata) and drawn in Arcmap 10.1 data view. The coordinates for the farms were projected in the map and pie charts created within the package.

The sampling effort was proportionally applied with the farm type strata to the districts according to the number of dairy farms, such that 46 (44%) farms were sampled in Mazabuka district of Southern Province, 32 (31%) farms were sampled in Chongwe, Kafue and Lusaka districts of Lusaka Province and 26 (25%) farms in Chibombo and Kabwe districts of Central Province, making a total of 104 farms (Table 1). The location of farms in the study site, the sample sizes on each farm and the proportions of the animals that tested positive and negative are given in Fig. 1.

### Prevalence of antimicrobial resistance

The population based design adjusted animal-level prevalence estimates of antimicrobial resistance *E. coli* (defined as resistant to at least one antibiotic) by farm type and overall are given in Table 2. The raw proportions (apparent prevalence) suggest a much higher prevalence in the commercial dairy farms (26.4%) compared to the small (12.7%) or medium scale farms (15.0%). However when the analysis takes account of the study design, there was no significant differences in true resistance prevalences across farm scales as judged by the overlap of the 95% CI’s (Table 2).

**Figure 2:**
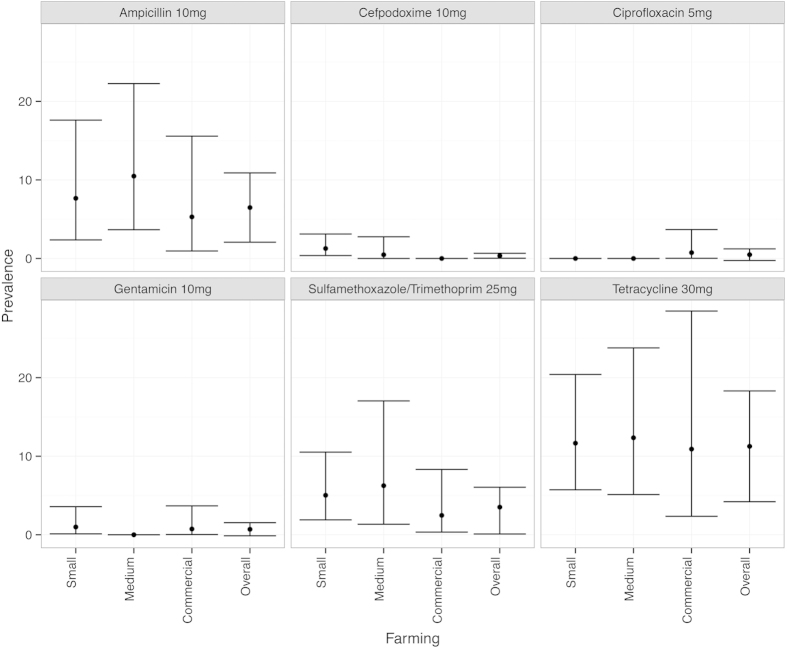
Prevalence of antimicrobial resistant *E. coli* to each antibiotic tested in relation to farming scale. Confidence intervals (95%) are depicted by the whiskers.

The results were further broken down and the population based design adjusted prevalence estimates for each antibiotic resistance type by farm type are plotted in Fig. 2. Resistance to tetracycline (Tet) was the most prevalent (overall 11.2%; 95%CI: 4.2–18.3) of the 6 antibiotics screened with little difference between the farm types. Resistance to ampicillin (Amp) was the second most prevalent (6.5%; 95% CI: 2.1–10.9) followed by sulfamethoxazole/trimethoprim (Sxt) and the prevalence of resistance to other three antibiotics, cefpodoxime (Cpd), ciprofloxacin (Cip) and gentamicin (Gen) was very low among the *E. coli* isolates obtained (Fig. 2).

### Phenotypic antimicrobial resistance patterns of E. coli isolates in the study sample

**Figure 3:**
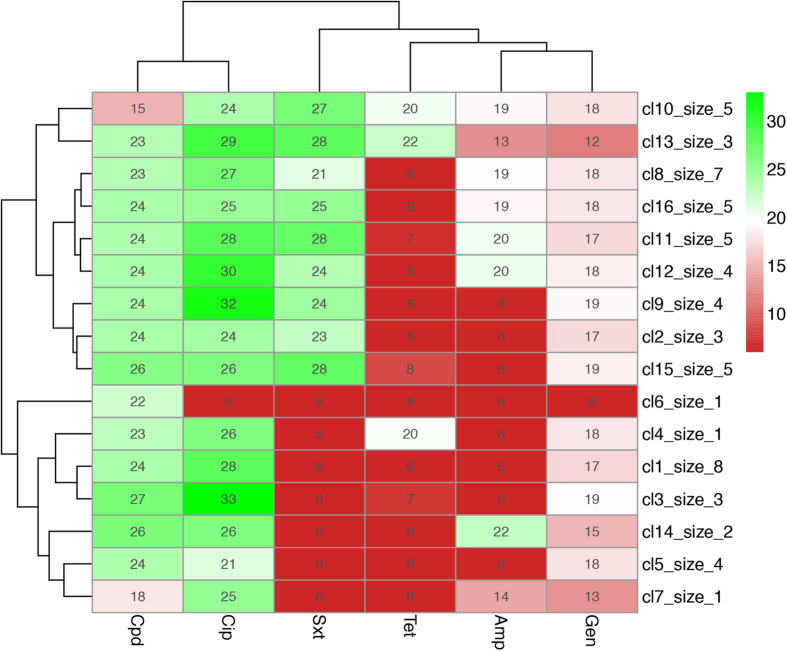
Patterns of antimicrobial resistance phenotypes for *E. coli* strains isolated in the study. The bottom matrix border shows the different types of antibiotics that were tested in the study, Cpd: cefpodoxime 10 mg; Cip: ciprofloxacin 5 mg; Amp: ampicillin 10 mg; Sxt: sulfamethaxazole/trimethoprim 25 mg; Tet: tetracycline 30 mg and Gen: gentamicin 10 mg. The colour indicates the susceptibility gradient with red indicating resistant and green indicating susceptible. The right margin indicates the antibiogram patterns of phenotypic resistance detected from 1 to 16 (cl1 to cl16) and the numbers of isolates in each resistance pattern from 1 to 10 (size_1 to size_10). The top margin dendrogram indicates the relationship among the antibiotics in terms of their susceptibility testing profiles. The dendrogram on the left margin indicates the clusters of antibiogram patterns in the study.

Sixteen antibiogram patterns of resistance were detected in this study (Fig. 3). The most prominent one was for the isolates that were resistant to Tet (n = 7). The second was for isolates that were resistance to a combination of TetAmp (n = 4). The third was resistance to SxtTet (n = 2). Pattern number four (n = 1) was noteworthy as it was resistant to all the antibiotics apart from Cpd. The dendrogram shows the clustering of antibiogram patterns for all the resistant isolates in Fig. 3. Resistance patterns based on disk diffusion tests were fully validated by MIC testing.

### Cattle diseases and usage of antibiotics

From the survey, cattle diseases were the main drivers for using antibiotics on the farms. The most common disease at farm level was mastitis with a prevalence of 100.0% (95%CI: 95.0–100.0) among the commercial, 77% (95%CI: 61.9–91.2) among the medium scale and 47% (95%CI: 37.99–56.78) at the small scale. Mastitis was mostly treated with an intra-mammary infusion (Mastiject®: tetracycline hydrochloride 200 mg, neomycin base 250 mg, bacitracin 2000 I.U, prednisolone 10 mg) with a disease episode treatment (DET) mean of 3.00 doses (95%CI: 2.17–3.83) per case in the commercial, 2.64 doses (95%CI: 1.60–3.67) for the medium scale farming and 0.61 doses (95%CI: 0.61–1.13) for the small farms.

Diarrhoea was the second most common disease in all three different farming scales with a prevalence of 77.8% (95%CI: 56.1–99.4) among the commercial, 64.8 (95%CI: 46.1–83.6) in the medium scale and 19.6% (95%CI: 11.7–27.5) among the small scale farms. The most commonly used treatments for diarrhoea were the injectable sulfa-based antibiotics such as sulfadimidine, sulfazine and trimethoprim sulphate with the DET mean of 3.00 injections (95%CI: 1.80–4.20) per case among the commercial farms, followed by 1.63 injections (95%CI: 0.35–2.86) among the medium scale and 0.22 injections (95%CI: 0.07–0.36) in the small scales farms. Other diseases were present at various levels in the different farm sizes as Supplementary Tables S1 and S2 online. It was found that intramuscular tetracycline was used as a specific treatment for theileriosis in combination with antiprotozoals. Penicillin with or without streptomycin was almost solely used for treatment of lumpy skin disease in combination with anti-inflammatory drugs. However, the two main antibiotics, tetracyclines and penicillins, were occasionally used in various combinations interchangeably across all the diseases.

### Multi-level logistic regression model for antibiotic resistance at the animal level

A univariate analysis of animal and farm level factors was carried out and all the factors with a p-value of less than or equal to 0.25 were passed forward for further analysis in a multivariate model. The final list of factors is listed in Table 3 with associated odds ratios and confidence intervals. The factors included: animals that were bred on the farm; treatment with tetracycline; treatment with any antibiotic; medium and commercial farms (big farms); Friesian and Jersey breeds (exotic breeds); presence of calves on the farm; lumpy skin disease (LSD); diarrhoea; foot rot; and the location of the farms (Table 3).

Breed of cattle was associated with an increased risk. Herds composed of only Jerseys and Friesian cattle were assigned with a new variable known as ‘Exotic herds’, as they are not indigenous cattle breeds. These herds were found to have an increased risk (OR = 2.89; 95%CI: 1.51–5.55) of antimicrobial resistance (Table 3). These animals may be more susceptible to local diseases, in addition to the fact that metabolically they have to divert more energy to production and may be more prone to diseases due to milk production stress.

The final model following forward and backward approaches included just an association with lumpy skin disease and the cattle age of more than 25 months. Farms that reported having had lumpy skin disease were more likely to have cattle that were positive for antimicrobial resistance (OR = 2.25; 95%CI: 1.03–4.91) (Table 4) even after adjusting for age which was associated with a protective effect with older age groups.

### Antibiotic sales

The pattern of resistance detected reflected the relative levels of sales of antibiotics, with the majority use of the tetracycline class matching the high prevalence of resistance found. A total of 41,280.87 kg of active ingredient of antibiotics was dispensed from the major supplier during the period under review (January 2013–February 2014). Of the total, 63.6% were parenteral antibiotic and 36.4% were oral ones. Tetracyclines were the most sold class of antibiotic during the period reviewed for both parenteral and oral sales and contributed 68% and 59% respectively (by weight). Penicillins were the second most abundant class of antibiotics sold by weight accounting for 27.2% of parenteral and 9.6% of oral sales. These were followed by the sulphonamides and then other class of antibiotics (see Supplementary Tables S3 and S4 online).

### Spatial clustering of the resistant E. coli isolates

There was no significant cluster of antimicrobial resistant *E. coli* detected in the study area using SATSCAN.

## Discussion

The aim of this study was to gain information on the use of antibiotics in small, medium and commercial dairy farms in central region of Zambia and subsequently determine the levels of antibiotic resistance to the commonly used veterinary antibiotics. The study made use of both questionnaires and resistance profile data from *E. coli* isolated from cattle. This allowed investigation of both drivers of antibiotic use and risk factors associated with the prevalence of resistance.

There was an apparent high prevalence of antimicrobial resistant *E. coli* in commercial farms when compared to the medium and small scale farms. Several of the large commercial farms were less willing to allow access and there was substantial variation in the proportion of resistant isolates detected on the commercial units (Table 2). As such the adjusted prevalence of antimicrobial resistance did not significantly differ across the three farm types, although practices in the commercial sector vary substantially. The commercial farms have predominantly pure breeds such as Holstein Friesian and Jerseys, referred to as ‘exotic breeds’ in this study. The main local breeds are Tonga, Baila and Angoni^8^; these have been crossed with the Holstein Friesian and Jersey breeds to improve milk production traits. These cross breeds were predominantly found among the small and medium scale farms. With the significant association between exotic breeds and isolation of resistant *E. coli*, future work is required to determine if local breeds are more resistant to specific diseases and therefore less antibiotics are used.

In this study, there was a specific correlation of antimicrobial resistant *E. coli* and the records of having lumpy skin disease on the farms. Lumpy skin disease was the only disease association with AMR from the multivariate analysis. Lumpy skin disease is highly infectious disease and as the skin lesions sometimes get secondary infections, farmers tend to use high doses of antibiotic treatments, mainly penicillin and/or oxytetracycline in affected animals and sometimes antibiotics in healthy animals with an apparent belief that they could be preventing the spread of this viral disease. In summary, farms that reported lumpy skin disease were more likely to have cattle with antimicrobial resistant *E. coli* in the gut than those which did not have the disease (Table 4).

A protective effect of older cattle (>25 months) for carriage of AMR isolates was also identified from the multivariate analysis and could be as a result of less treatment of this group of animals when compared to young animals (<25 months) which are more prone to diarrhoea and respiratory diseases resulting in more use of antibiotics. This observation is in line with other studies that indicated that AMR is more prevalent in younger cattle than in the older ones^7^.

There were more sales of tetracycline than any other antibiotics at the main supply depot in Lusaka and this was also reflected in the responses from the farmers and the level of resistances detected in *E. coli*. Although tetracycline was used as a treatment for all diseases, it is important to note that it is used prophylactically and therapeutically alone or in combination with an antiprotozoal such as buparvaquone to prevent and treat *Theileria* infection. *Theileria* parasite growth is inhibited by tetracycline. Theileriosis occurs sporadically throughout the year with outbreaks mostly associated with the rainy seasons (December to February) and during the dry months (May to July)^9^,^10^ and is a major disease resulting in high mortality of cattle in the study area. This survey was conducted in the off-season and so was not co-incident with Theileriosis, and this may explain why treatment for *Theileria* was not identified as a risk factor for carriage of antibiotic resistant *E. coli* in the final model.

The lack of correlation with mastitis, the most common disease, may reflect the direct administration of antibiotics into the mammary glands, where the active ingredients are concentrated in the udder, thereby providing limited opportunity for resistance to be selected for in commensal intestinal *E. coli*.

In general, the prevalences of antibiotic resistance in this study were lower when compared to antimicrobial resistance reports from other parts of Africa^11^ and other continents^12^,^13^. The prevalences will differ due to sampling study design and estimation method or if true may be attributable to a wide range of factors including economic prosperity and affordability as we found that some of the reported diseases were left untreated, possibly due to the cost of purchasing antibiotics. Most of the farmers (91%) purchased the antibiotics from established small or big veterinary drugs shops. The fact that most of the antibiotics used in the study area were sourced from well-established suppliers, and thus likely to be of proven provenance, is important as this reduces the potential risk of antimicrobial resistance emerging from the use of poorly formulated products. However, a substantial number of responders (35%) indicated that prescriptions were not asked for by the retailers which does indicate the lack of a strong inspectorate system around the sale of antibiotics in Zambia despite having laws and regulations in place.

Sixteen distinct AMR patterns were observed based on the six antibiotics tested. Some groupings were more common than others, indicating co-selection of resistance alleles (Fig. 3). Future work will examine the genetic basis of resistance in these strains and establish whether particular alleles are associated, for example on plasmids. Sequencing will also allow fine mapping of the distribution of antibiotic resistance alleles in the region and if sequence data can be obtained from human isolates, then insight the possible transmission of alleles between humans and cattle can be established.

Overall, the study has produced a general picture of the use of antimicrobials in the dairy sector in the region of Zambia studied. More access to commercial units would have been desirable and the very wide confidence intervals for the commercial farms tested may indicate quite distinct practices. The study indicates that by working with mixed breeds and not pure imported breeds might provide a basis to more sustainable dairy farming in these settings. Future work will address resistance allele distribution in cattle and human isolates in the study area.

## Materials and Methods

### Study design and sampling

The target population was dairy cattle on farms in the central region of Zambia, within a 120 km radius from Lusaka district, covering parts of Lusaka, Central and Southern Provinces. A sampling frame of all known dairy herds was developed from records held by the District Veterinary Officers (DVOs) and the management of the Milk Collection Centres (MCC). Based on local knowledge and the census data from the sampling frame, herds were stratified into small (1–20 cattle), medium (>20 cattle) and commercial units. Medium and commercial farms were subjectively classified based on levels of production mechanisation and clear commercially orientated activities such as milk packaging or/and processing, use of paper records or electronic data bases. Medium-sized farms had well defined farm entrepreneurship and management systems but less developed when compared to commercial farms.

Parameter estimates used to calculate the sample size of the study were derived from the analysis of 81 samples, collected from 12 farms during a pilot study in June and July 2013. The prevalence of antibiotic resistance at the animal level in the pilot study was 10%, the intraclass correlation coefficient (ρ) showed more variability between farms (ρ = 0.027) when compared to the within farms (ρ = 0.112). Sample size was calculated using Epitools-Ausvet®^14^ with the following assumptions: sampling 1 animal per farm; 10% of animals have antibiotic resistance isolates of *E. coli*; a perfect test; a 5% level of precision; and 95% level of confidence. A total of 110 farms was desired, however, to capture more of the variation it was decided to sample 3 animals per epidemiological unit (defined as a number of animals managed as a group) on each farm; these were randomly selected without replacement from a list of animals using random number tables. The herds were sampled proportionally by the numbers in each of the three categories in the sampling frame and then proportionally to the geographic distribution based on the government administrative boundaries to ensure a good geographical spread. Seventy-five per cent of herds were small, 20% were medium and 5% commercial. The target sample size was then 83, 17 and 4, respectively. However, as the number of commercial farms was very small in the sample and they were more accessible, it was decided to aim to sample all 26. Thus a stratified, multistage random sampling of cattle with unequal probabilities was generated (Table 1).

### Farm data collection

Two types of questionnaires were designed and used to collect data at both the individual animal and farm levels. For individual cattle the following information was collected; animal ID, lactation status, breed, sex, age, health status, whether or not it was born on the farm or sourced from another farm and whether it had received antibiotic treatment in the twelve months preceding the study period. At the farm level, information was obtained on farm management and the use of antibiotics.

Four research assistants were trained by the principal investigator to help in questionnaire administration and faecal sample collection.

### Faecal sample collection and E. coli isolation

Faecal samples were collected per rectum using a gloved hand. A fresh glove was used for each animal. Each glove was inverted and sealed immediately after the collection of faecal material. In instances where the rectum was empty, the animal was stimulated to defecate. Faecal samples were kept on ice in a portable refrigerator (ADDIS®, South Africa) connected to the vehicle and transported to the laboratory in Lusaka within 48 hours of collection. The samples were enriched in Buffered Peptone Water in order to help recover *E. coli.* From each faecal sample, 10 g was mixed with 90 ml of Buffered Peptone Water (BPW)(Oxoid, UK) and incubated overnight statically at 37 °C. The enrichments were spread with a sterile swab onto lactose MacConkey agar (Oxoid, UK) and these were incubated for 16 to 18 hours (overnight) at 37 °C. One colony from each plate suspected to be *E. coli*, based on appearance, was picked and streaked onto Tryptone Bile-X-Glucuronide (TBX) agar plates (Oxoid, UK) and incubated for 16 to 18 hours at 37 °C. Blue colony colouration as a result of glucuronidase production was taken as an indication that the colony was *E. coli*. It was noted from subsequent phylogenotyping^15^ of the 371 isolates by PCR, that only 10 (3%) could not be typed indicating that >95% of the isolates studied were *E. coli* (data not shown). All faecal sampling and animal handling of the farm animals was carried out in accordance with the approved guidelines issued by The Roslin Institute Animal Welfare and Ethical Review Body which approved this study.

### Antimicrobial Susceptibility Testing (AST)

The AST was carried out using the Kirby-Bauer disc diffusion method^16^. For each isolate, one colony from the TBX plate was streaked onto a non-selective blood agar plate. One colony from the blood agar plate was used to inoculate 10 ml of nutrient broth and statically incubated for 18–24 hours (overnight). About 600 μl–1000 μl from each suspension of overnight nutrient broth culture was adjusted with 5 ml of sterile saline to match the visual opacity with that of 0.5 a McFarland standard, containing approximately 1–2 × 10^8^ colony forming units (CFU)/ml of the *E. coli* strain American Type Culture Collection (ATCC) 25922. The saline suspension was spread onto the surface of a Mueller-Hinton Agar plate with a sterile swab (Oxoid, UK). The antimicrobial discs (Oxoid, UK) containing antibiotics of both veterinary and human importance (tetracycline 30 mg, ampicillin 10 mg, sulfamethoxazole/trimethoprim 25 mg, gentamicin 10 mg, cefpodoxime 10 mg, and ciprofloxacin 5 mg) were dispensed onto the surface of the Mueller Hinton Agar plates at least 24 mm apart from the centre of each other using a Multi-disc dispenser (Oxoid, UK). The plates were incubated at 37 °C for 16–18 hours. The diameters of the inhibited zones were measured; including the diameter of the discs to the nearest whole millimetre using sliding callipers and interpreted using standard break points^17^.

The minimal inhibitory concentration (MIC) for all six antibiotics for the majority of the resistant *E. coli* isolates was assessed using an E-strip method^18^ for resistance confirmatory purposes. The inoculum was spread onto Mueller Hinton Agar plates (Oxoid, UK) as above. The specific E—strip containing the antibiotic of interest was placed on the plate and incubated for 16–18 hours before visually taking the readings. *E. coli* strain ATCC 25922 was used for reference in all the culture assays.

### Data management

Questionnaire data and all diagnostic results from culture and typing were transferred from paper working copies to an Access (Microsoft 2010) database. The data on the sales of antibiotics were collated in Excel (Microsoft 2010) from the antibiotic supplier. The data was screened using summary tables and univariate plots for missing and unexpected values, and where possible these were resolved by referring back to the paper records.

### Statistical analysis

Population based prevalences were estimated using the *survey* package^19^ in R software environment version 3.1.1 (http://cran.r-project.org/). In order to account for the sampling method that was used of unequal probability to size, statistical weights were used in the analysis. From the final dataset individual herd weightings were generated based on the total number of herds in a given district of a particular category divided by the final number actually sampled. For example in Chibombo (Chb) 56 smallholdings were present of which 12 were sampled so the weighting at the herd level was 56/12. In a similar way, individual animal weightings were calculated from the total number of animals present on the farm divided by the sample size taken on that farm. The final animal weight was then the product of the herd and animal level weightings and is applied to data such as prevalence of resistance to allow comparisons across farms and districts. These weightings were used in the creation of a design object that the *survey* package can use to adjust prevalence estimates.

A standard multi-level logistic regression model was developed with antibiotic resistance status to one or more antibiotics as a binary outcome. The model was developed using the standard Hosmer Lemeshaw^20^ approach where first potential explanatory variables or risk factors were screened and any variables with a p-value < 0.25 from the Wald statistic were allowed to pass for use in a multivariate multi-level model. The model was built using the *lme4* package in R^21^. Both forward and backward approaches were explored with variables being entered or removed based on their Wald statistic p-value. As variables were added or removed they were tested using a likelihood ratio test of the change in deviance and variables that were significant at the 5% level were retained. Colinearity of all potential explanatory variables was checked using the Pearson rank correlation statistic.

Diagnostics were performed and plots of residuals were examined, confirming the goodness-of-fit of the model. Odds ratios (OR) and their associated 95% confidence intervals were estimated in the final logistic model for factors statistically significantly associated with the presence of antibiotic resistant *E. coli* in individual cattle.

The distribution of resistant *E. coli* on farms was examined to determine if they occurred randomly or if there was some clustering. SATSCAN® was used to search the significant clusters of antimicrobial resistant *E. coli*. We used Bernoulli algorithm model, involving geometry of the study area, the probability distribution of the events and different shapes and sizes of the window to scan through possible cluster in the sample (Kulldorff, M. Satscan user guide. *StatScan, Boston, USA* (2006). The locations of farms were mapped using ARCGIS® (ESRI, ArcGIS desktop: release 10. *Environmental Systems Research Institute, CA* (2011)).

## Additional Information

**How to cite this article**: Mainda, G. *et al.* Prevalence and patterns of antimicrobial resistance among *Escherichia coli* isolated from Zambian dairy cattle across different production systems. *Sci. Rep.* **5**, 12439; doi: 10.1038/srep12439 (2015).

## Supplementary Material

Supplementary Information

## Figures and Tables

**Table 1:** Summary of the study sampling frame; including number of herds of each type; the number of herds sampled by farm type & district; the number of animals sampled on those farms.

**Districts**																		
Farming scale	**Chibombo**			**Chongwe**			**Kabwe**			**Kafue**			**Lusaka**			**Mazabuka**		
	**N**	**n**	**s**	**N**	**n**	**s**	**N**	**n**	**s**	**N**	**n**	**s**	**N**	**n**	**s**	**N**	**n**	**s**
Small	56	12	34	59	12	36	22	5	15	29	6	18	0	0	0	217	42	126
Medium	9	2	6	15	2	9	17	3	9	19	4	12	19	2	15	20	3	9
Commercial	2	1	9	8	3	18	3	3	18	0	0	0	7	3	27	6	1	15
Total	67	15	49	82	17	63	42	11	42	48	10	30	26	5	42	243	46	150

N: Total number of farms of a given category in the district; n: number of farms sampled; s: number of animals sampled.

**Table 2:** Prevalence of antibiotic resistant *E. coli* by farm scale.

**Farming**	**No. resistant animals/total**	**Population based design adjusted prevalence**
Small	29/226	13.7 (7.4–22.6)
Medium	9/59	13.0 (5.6–24.2)
Commercial	23/86	11.7 (2.5–30.0)
Overall	61/371	12.3 (4.7–19.8)

**Table 3:** Univariate analysis for risk factors associated with detection of antibiotic resistant *E. coli*.

**Factor**	**Unit**	**Odds Ratio (95%CI)**	**P**
Cattle bred on-farm	Yes	2.13 (0.98–5.33)	0.077
Tetracycline treatment	Yes	0.41 (0.10–1.20)	0.154
Any antibiotic treatment	Yes	0.63 (0.28–1.30)	0.235
Commercial	Yes	2.04 (0.77–5.41)	0.103
Exotic herds	Yes	2.89 (1.51–5.55)	<0.001
Cows*	Number	1.48 (1.04–2.07)	0.025
Calves*	Number	1.78 (1.15–2.74)	0.008
Age	>25months	0.24 (0.13–0.44)	<0.001
Lumpy Skin Disease	Presence	2.14 (1.13–4.03)	0.019
Diarrhoea	Presence	1.62 (0.78–3.33)	0.192
Lactating	Yes	0.75 (0.41–1.33)	0.048
Contact with other herds	Yes	0.67 (0.35 -1.22)	0.206
Foot rot	Presence	2.12 (1.17–3.79)	0.012
Lusaka district	Yes	5.84 (1.63–20.9)	0.006

^*^Figures were log10 transformed.

**Table 4:** Multi-level logistic regression model for risk factors associated with detection of antibiotic resistant *E. coli*.

**Risk factor**	**Estimate (95%CI)**	**P**
Lumpy skin disease	2.254 (1.034–4.908)	0.014
Cattle (>25 months)	0.314 (0.139–0.707)	0.001
