# Erratum: Prevalence and patterns of antimicrobial resistance among *Escherichia coli* isolated from Zambian dairy cattle across different production systems

**DOI:** 10.1038/srep14162

**Published:** 2015-09-21

**Authors:** Geoffrey Mainda, Paul R. Bessell, John B. Muma, Sean P. McAteer, Margo E. Chase-Topping, James Gibbons, Mark P. Stevens, David L. Gally, Barend M. deC. Bronsvoort

Scientific Reports 5: Article number: 1243910.1038/srep12439; published online: 07272015; updated: 09212015

The original version of this Article contained an error in the spelling of the author Paul R. Bessell, which was incorrectly given as Paul B. Bessell. This has now been corrected in both the PDF and HTML versions of the Article.

