# Supplementary material for: Prevalence and patterns of antimicrobial resistance among Escherichia coli isolated from Zambian dairy cattle across different production systems
Source: Sci Rep. 2015 Jul 27;5:12439. doi: 10.1038/srep12439 (PMC4515737; doi:10.1038/srep12439)
Supplement: Supplementary Information [file srep12439-s1.pdf]

Prevalence and patterns of antimicrobial resistance among *Escherichia coli* isolated from Zambian dairy cattle across different production systems

SUPPLEMENTARY INFORMATION

***Geoffrey Mainda[1,3], Paul Bessell[2], John B. Muma[4], Sean P. McAteer[1], Margo E. Chase-Topping[5], James Gibbons[6], Mark P. Stevens[1], David L. Gally[1] and Barend M. deC. Bronsvoort[1,2]**

Divisions of Immunity and Infection[1] & Veterinary Clinical Sciences[2], The Roslin Institute and The Royal (Dick) School of Veterinary Sciences, The University of Edinburgh, Easter Bush, Midlothian, EH25 9RG, United Kingdom

[3]District Veterinary Office, Ministry of Agriculture and Livestock, P.O. Box 80285, Kabwe, Zambia

[4]Department of Disease Control, School of Veterinary Medicine, University of Zambia, Zambia

[5]Centre for Immunity, Infection and Evolution, Kings Buildings, University of Edinburgh, Charlotte Auerbach Road, Edinburgh, EH9 3FL, United Kingdom

[6]School of Veterinary Medicine, Veterinary Science Centre, Belfield, Dublin 4, Eire

***Communicating author: Geoffrey Mainda, Division of Immunity and Infection The Roslin Institute and R(D)SVS, University of Edinburgh, Edinburgh, EH25 9RG, UK.**

Tel: 00 44 131 6519231.

Email: geoffrey.mainda@roslin.ed.ac.uk; geoffreymainda@yahoo.co.uk

Supplementary Table S1: Means percentages of number of farms with standard error of mean (SEM) on the presence/absence of specific diseases/syndromes and number of farms which did not provide information (Unknown).

		Presence		Absence		
Category (n= Total farms)	Disease/ Syndrome	Farm (%)	SEM	Farm (%)	SEM	Unknown
Small (n=77)	Mastitis	47	5	53	5	2
	Diarrhoea	40	4	80	4	1
	Lumpy Skin Disease	25	44	73	44	2
	Post parturient	10	3	90	3	3
	Tick-borne	15	38	86	37	*3*
	Foot rot	11	3	89	3	3
	Respiratory	10	3	90	3	3
	Digestive	5	2	95	2	3
	Others	19	5	81	5	3

Supplementary Table S2: Means percentages of number of farms with standard error of mean (SEM) on the presence/absence of specific diseases/syndromes and number of farms which did not provide information (Unknown).

		Presence		Absence		
Category	Disease/Syndrome	Farm (%)	SEM	Farm (%)	SEM	Unknown
Medium (n=16)	Mastitis	77	7	23	7	1
	Diarrhoea	64	10	35	10	1
	Lumpy Skin Disease	45	15	55	15	1
	Post parturient	38	15	62	15	1
	Tick-borne	11	7	89	6	*1*
	Foot rot	25	14	75	14	1
	Respiratory	37	8	63	8	1
	Digestive	45	15	55	15	1
	Others	55	10	45	10	1
Commercial (n= 11)	Mastitis	100	0	0	0	2
	Diarrhoea	78	11	22	11	3
	Lumpy Skin Disease	25	12	75	12	3
	Post parturient	63	14	38	14	3
	Tick-borne	26	12	76	12	*3*
	Foot rot	67	13	33	13	3
	Respiratory	38	14	63	14	3
	Digestive	62	14	38	14	3
	Others	33	13	67	13	3

Supplementary Table S3: Parenteral antibiotics

Antibiotic	Quantity(kg)	Percentage (%)
Tetracycline	17,840.20	68.00
Penicillin	7,139.00	27.21
Sulphonamide	739.90	2.82
Aminoglycoside	337.00	1.28
Tylosin	98.00	0.37
Floroquinolones	80.30	0.31
Macrolide	2.00	0.01

Supplementary Table S4: Oral antibiotics

Antibiotic	Quantity(kg)	Percentage (%)
Tetracycline	9,177.22	61.00
Sulphonamide	2,790.40	18.55
Penicillin	1,508.00	10.02
Nitrofurans	695.23	4.62
Fluroquinolone	439.00	2.92
Polymyxin	266.00	1.77
Pleuromutilin	89.00	0.59
Tylosin	79.62	0.53
